# Analyses of All Small Molecule-Based Pentacene/C_60_ Organic Photodiodes Using Vacuum Evaporation Method

**DOI:** 10.3390/nano13212820

**Published:** 2023-10-24

**Authors:** Young Woo Kim, Dongwoon Lee, Yongmin Jeon, Hocheon Yoo, Eou-Sik Cho, Ezgi Darici, Young-Jun Park, Kang-Il Seo, Sang-Jik Kwon

**Affiliations:** 1 Department of Electronics Engineering, Gachon University, 1342 Seongnam-Daero, Sujeong-gu, Seongnam City 13120, Gyeonggi-do, Republic of Korea; mae04008@gachon.ac.kr (Y.W.K.); grw04214@gachon.ac.kr (D.L.); hyoo@gachon.ac.kr (H.Y.); es.cho@gachon.ac.kr (E.-S.C.); 2 Department of Biomedical Engineering, Gachon University, 1342 Seongnam-Daero, Sujeong-gu, Seongnam City 13120, Gyeonggi-do, Republic of Korea; yongmin@gachon.ac.kr; 3 CLAP Co., Ltd., 1342 Seongnam-Daero, Sujeong-gu, Seongnam City 13120, Gyeonggi-do, Republic of Korea; ezgi.darici@clap.co.kr (E.D.); yjpark@clap.co.kr (Y.-J.P.); kiseo@clap.co.kr (K.-I.S.)

**Keywords:** OPDI, small molecules, pentacene, C60, vacuum processing, cavity effect

## Abstract

The vacuum process using small molecule-based organic materials to make organic photodiodes (OPDIs) will provide many promising features, such as well-defined molecular structure, large scalability, process repeatability, and good compatibility for CMOS integration, compared to the widely used Solution process. We present the performance of planar heterojunction OPDIs based on pentacene as the electron donor and C60 as the electron acceptor. In these devices, MoO_3_ and BCP interfacial layers were interlaced between the electrodes and the active layer as the electron- and hole-blocking layer, respectively. Typically, BCP played a good role in suppressing the dark current by two orders higher than that without that layer. These devices showed a significant dependence of the performance on the thickness of the pentacene. In particular, with the pentacene thickness of 25 nm, an external quantum efficiency at the 360 nm wavelength according to the peak absorption of C60 was enhanced by 1.5 times due to a cavity effect, compared to that of the non-cavity device. This work shows the importance of a vacuum processing approach based on small molecules for OPDIs, and the possibility of improving the performance via the optimization of the device architecture.

## 1. Introduction

The organic photodiode (OPDI) is one of the photodetectors (PDs) made with organic semiconductor materials to convert incident photons into an electrical signal. The PDs have been extensively studied and developed due to their promising applications, such as image sensing, health monitoring, night vision, and environmental monitoring [1,2,3,4,5,6,7]. Of those PDs, organic photodiodes provide unique advantages by virtue of the realization of flexible, lightweight, and wearable devices. To date, the device structures consisting of OPDIs have been made with both polymers and small molecules. The small molecule-based vacuum deposition techniques have already been used for the organic light-emitting diode (OLED) commercial industry. Thus, the vacuum process techniques using small molecules are a potential candidate for the OPDIs industry, compared to a polymer-based solution process. For several performances of the OPDIs, the most important characteristics are the responsivity (R) and dark current (J_D_) in the reverse bias [8,9,10,11,12]. The responsivity is defined as the ratio of the photocurrent to incident power (A/watt). For linear photodetectors, the responsivity is a function of the wavelength of the incident radiation. For solution-processed OPDIs, many efforts have been made to suppress the dark current density in the nano ampere (nA) range in the reverse bias region [13,14,15,16]. The vacuum deposition process provides further benefits, such as well-defined molecular structure, high purity, large scalability, and good process repeatability [17,18]. However, an important factor to achieve successful OPDIs using the small molecule-based vacuum process is the power conversion efficiency (PCE), that is, the external quantum efficiency (EQE).

In organic semiconductors structured by weak Van der Walls forces, the electrical properties are mainly co-related to the energy levels of the lowest unoccupied molecular orbital (LUMO) and the highest occupied molecular orbital (HOMO), unlike the energy bands in inorganic semiconductors. It is well known that excitons are created due to a strong Coulombic interaction of electron–hole pairs generated by photon absorption. To obtain a photocurrent, the excitons should be diffused to the interfacial layer between electron-donating (p-type) and electron-accepting (n-type) materials, and separated by virtue of a driving force created from the difference of electron affinities [19,20,21]. For efficient photocurrent generation, the structure of active layers consisting of donor and accepter materials can be divided into the planar heterojunction (PHJ) and the bulk heterojunction (BHJ).

In this study, we sought to fabricate OPDIs with all small molecular PHJ layers using the vacuum evaporation process. The vacuum evaporation technique for all small molecular OPDIs offers a promise of compatibility with other circuitry, such as CMOS and OLEDs, large scalability, and batch-to-batch repeatability. The device has a structure of active layers with pentacene as the donor and C60 as the acceptor. The Pentacene/C60 planar heterojunction was fabricated with the structure of a photo diode consisting of ITO (150 nm)/MoO_3_ (10 nm)/Pentacene (45 nm)/C60 (50 nm) /bathocuproine (BCP) (10 nm)/ Al (100 nm). MoO_3_ and BCP as interlayers were applied to act as an electron-blocking layer (EBL) and hole-blocking layer (HBL), respectively. The blocking layers can prevent charge injection from the electrodes under reverse bias. To investigate the proper thickness of the absorbing layer, the pentacene layer was varied, and the J–V characteristics were compared. In addition, the effects of EBL and HBL on the J–V characteristics were analyzed. Absorption spectra were analyzed for each active layer by UV–Vis spectrophotometry. 

## 2. Experimental Methods

An OPDI with a Pentacene/C60 PHJ structure was fabricated using a vacuum evaporation system. The vacuum evaporation system consists of the bichambers of both an organic chamber and a metal chamber. The base vacuum of 1.7 × 10^−7^ torr was obtained using a cryo pump. Glass substrates with an active area of 1.5 mm × 1.5 mm defined by a pixel definition layer (PDL) on a pre-patterned ITO anode were sequentially cleaned in an ultrasonic bath in acetone for 10 min and isopropyl alcohol (IPA) for 10 min, and deionized water for 10 min. After blow-drying with a nitrogen gun, the substrates were dried on a hot plate at 150 °C for 5 min. Finally, the substrates were placed in a frame holder and loaded into the organic chamber. Molybdenum oxide (MoO_3_) was evaporated with 10 nm as an EBL. Pentacene (donor, 45 nm) and C60 (acceptor, 50 nm) were sequentially evaporated as an active layer. Bathocuproine (BCP, 10 nm) was evaporated as an HBL, the substrate was moved into the metal chamber without breaking vacuum, and aluminum (100 nm) was evaporated as a cathode layer. The fabricated OPDI device was unloaded into a glove box filled with dry nitrogen gas and encapsulated with a glass plate using a UV-curable epoxy resin.

To analyze the influence of the EBL and HBL on the dark current, several different types of devices were fabricated. That is, the device without EBL (MoO_3_), the device without HBL (BCP), and the device without EBL and HBL were prepared. In this paper, we refer the whole device structure with EBL and BHL to a standard structure.

The dark current was measured using an HP4156C source-meter with the sample mounted in the dark box probe station. To quickly analyze the response to light illumination, Halogen lamp luminance and Spectrometry were used with three illumination levels of low, medium, and high at (579.9, 1555.0, and 9791.6) W/m^2^, respectively. To optimize the device performance, the thickness of the p-type layer or electron donor layer (pentacene) was varied (0 to 100) nm, and the external quantum efficiency was measured using QE400 NIR (TNE Tech Co., Ltd., Yongin-si, Republic of Korea), consisting of a 300 W Xenon light source, optical chopper at (4–99) Hz, monochromator, custom designed current amplifier, and digital lock-in amplifier (4-Ch 24-Bit Resolution), with a measurement wavelength range (250–1700) nm.

## 3. Results and Discussion

### 3.1. J–V Characteristics Depending on the Interfacial Layers

**Figure 1:**
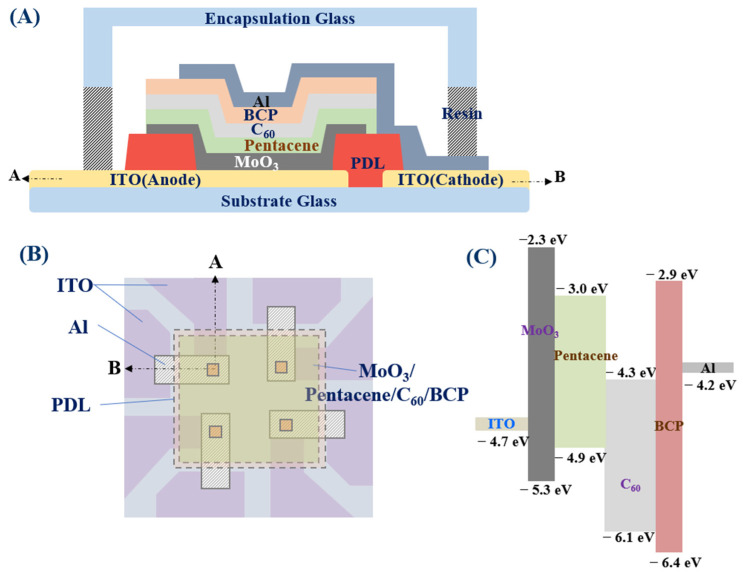
(**A**) The standard device structure, (**B**) layout, and (**C**) schematic of the estimated energy.

**Figure 2:**
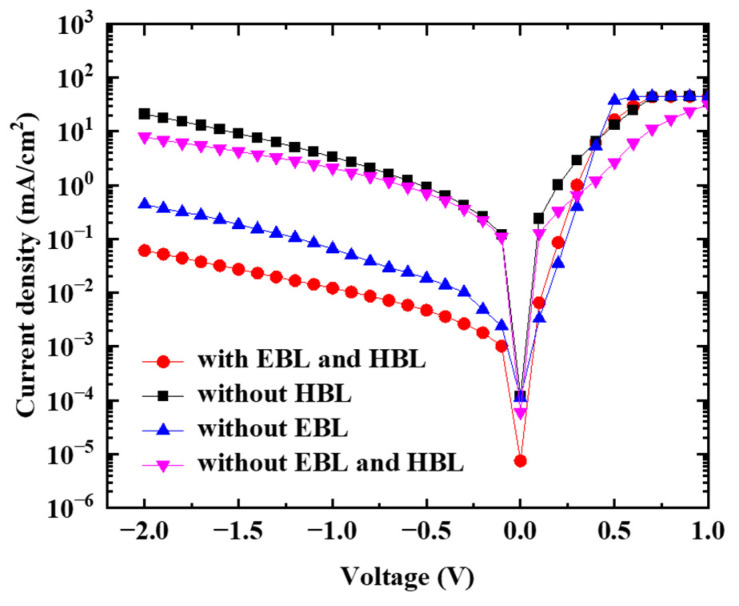
The dark current density–voltage (J–V) characteristics for the four types of devices, including the standard structure with the two interfacial layers of EBL and HBL.

Figure 1A shows the standard device structure, Figure 1B shows the planar layout design, and Figure 1C shows a schematic of its estimated energy level. Figure 2 shows the dark current density–voltage (J–V) characteristics for the four types of devices, including the standard structure with the two interfacial layers of EBL and HBL. The influence of the blocking layers on the dark current becomes apparent in the J–V characteristics. The standard device structure exhibits a dark current density of 0.013 mA/cm^2^ at −1 V reverse bias. The OPDI devices without EBL, and without both EBL and HBL, show the dark current of (0.066 and 2.068) mA/cm^2^, respectively, at −1 V reverse bias. Meanwhile, the OPDI device without HBL shows the dark current of 3.347 mA/cm^2^ at −1 V reverse bias, which is a factor of 279 higher than that for the standard device. The results show that the HBL dominantly affects the dark current. Without the BCP HBL, the dark current density increases rapidly as the reverse bias increases, and the degree of the increased dark current is more severe than that of the device without both EBL and HBL. It is estimated that the holes injected into the active layer from the Al electrode without HBL can transport to the ITO/MoO_3_ layers and accumulate at the MoO_3_/Pentacene interface. The accumulated holes create a strong localized electric field at the MoO_3_/Pentacene interface, and result in the injection of electrons from the ITO anode, enhancing the dark current [22]. A low saturation current under reverse bias is an important requirement of the OPDI, and thus, the HBL and EBL interfacial layers in this device architecture are necessary. The thicknesses of the MoO_3_ and BPL block layers were fixed according to the most proper thicknesses (10 nm) with making reference to the literature because it should take too much time for optimizing each thickness. Thus, there will be room for obtaining the optimum figure of merit of the OPDI performance in terms of the dark current and the efficiency [23]. 

**Figure 3:**
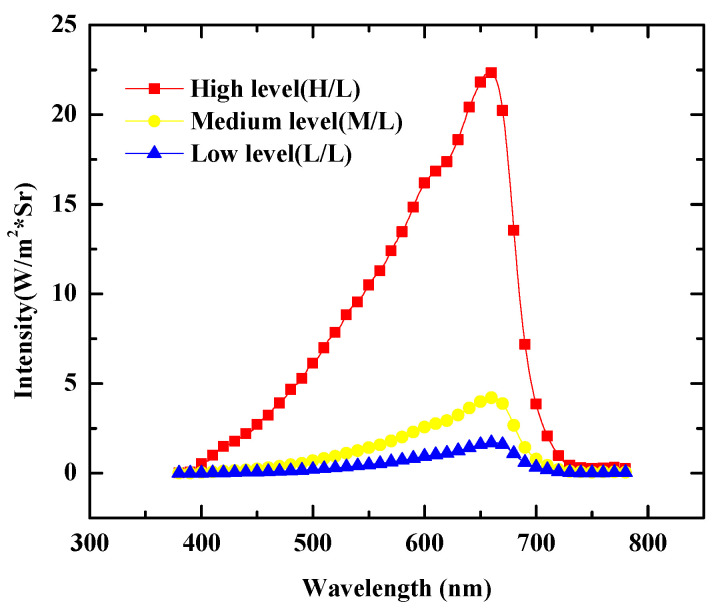
The luminance spectrum of the Halogen lamp light source used with three illumination levels of low, medium, and high at (579.9, 1555.0, and 9791.6) W/m^2^, respectively.

**Figure 4:**
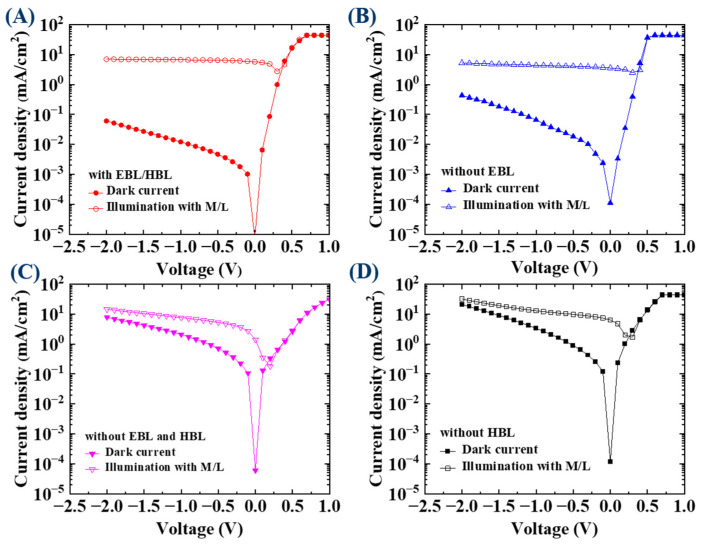
J–V characteristics under the illumination of the medium level compared with the dark current for the device (**A**) with EBL and HBL, (**B**) without EBL, (**C**) without EBL and HBL, and (**D**) without HBL.

**Figure 5:**
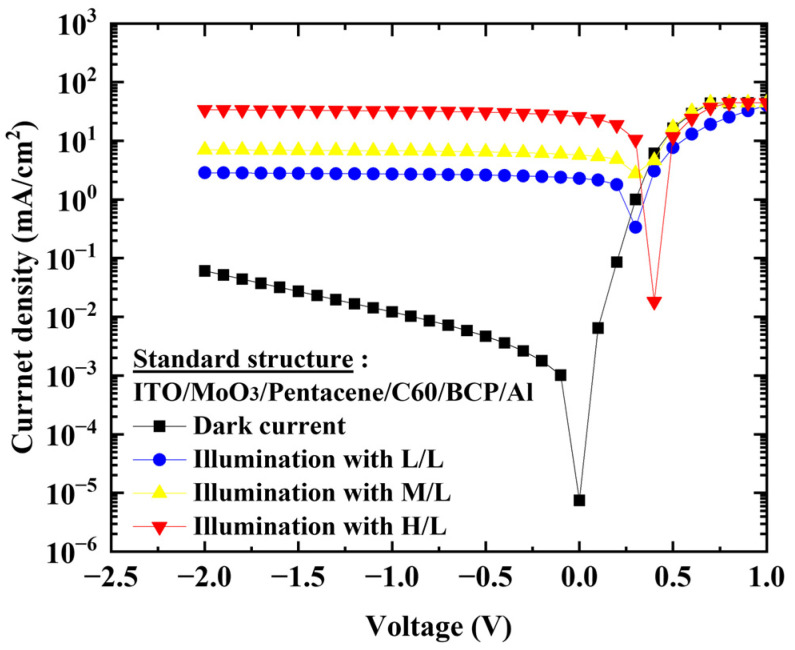
J–V characteristics measured at the three illumination levels for the standard OPDI with HBL and EBL.

To quickly analyze the J–V characteristic under illumination, a Halogen lamp light source was used and the luminance spectrum is shown in Figure 3 with three illumination levels of low, medium, and high at (579.9, 1555.0, and 9791.6) W/m^2^, respectively. For the four types of structures, the J–V characteristics were measured under the illumination of the medium level (1555.0 W/m^2^) and compared with the dark current density, as shown in Figure 4. When both the HBL and EBL were used, the ratio of on-current (6.7 mA/cm^2^) to dark current (12.1 × 10^−3^ mA/cm^2^) under a reverse bias of −1 V was about 553:1, while when the HBL was omitted, the ratio was about 3.9:1 ((12.9 to 3.3) mA/cm^2^). Table 1 summarizes these results. Moreover, when both the HBL and EBL were used, the photocurrent level was saturated independently of the reverse bias. However, when the HBL was not used, the photocurrent was not saturated, and was affected by the dark current. For the standard OPDI with the HBL and EBL, the J–V characteristics were measured at the three illumination levels, and Figure 5 shows the results. The photocurrent responds well to the illumination levels and becomes saturated for all of them. The saturation of the current level under reverse bias is a very important requirement for the OPDI operation. It is notable that MoO_3_ and BCP play a good role in blocking layers and contribute to the saturated operation in this OPDI architecture.

### 3.2. Device Performance with Variable Active Layer Thickness

In terms of the dark current and the external quantum efficiency, the thickness of the active layer plays a critical role. The dark current is a critical parameter determining the specific detectivity, which is important for the light detection function of the OPDI. Depending on the material properties and device structures, the dark current range can span several orders [24,25]. To suppress the dark current in organic diodes, several approaches have been reported in the literature [14,26,27,28]. One of the most frequently used methods is the suppression of shunt paths [29]. There are several sources of the dark current of the OPDIs, however, this remains controversial due to lack of comprehensive understanding [25,30]. The intrinsic dark current typically comprises a thermal generation of charges over the gap within the active layer, and a charge carrier injection from the metal contact into the organic semiconductor. However, in practical organic diodes, charge-transfer (CT) states are present at the donor–acceptor interface [31,32], and the effective gap for thermal generation, the characteristic charge-transfer state energy (E_CT_), is usually lower than the gap of the single organic semiconductor. In addition, trap states with intra-gap energies in real organic semiconductors are taken into account, usually due to the disordered nature of the organic materials, structural defects of the devices, and impurities present in the materials [33,34]. Several reports mention the influence of the trap states on the limited charge transport, increased recombination rate, and increase in the dark current [35,36,37,38].

**Figure 6:**
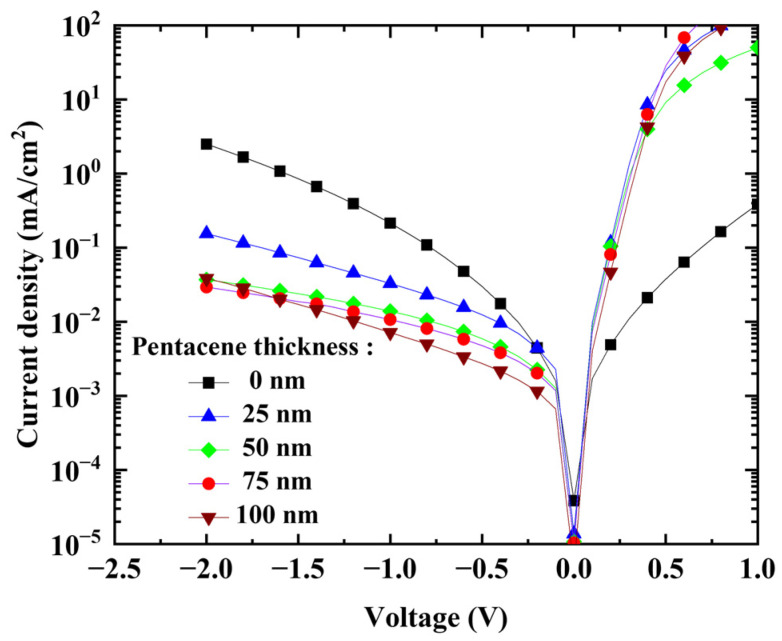
J–V characteristics with pentacene thickness measured under dark condition.

In this study, the thickness of the p-type electron donor layer (pentacene) was varied from (25 to 100) nm. Figure 6 shows the current density–voltage characteristics under the dark condition. The dark current decreased as the thickness of the pentacene layer increased, and this may have resulted from the higher shunt resistance due to the thicker layer, and as a result, the effective suppression of electron injection by the electron-blocking layer [39,40,41].

**Figure 7:**
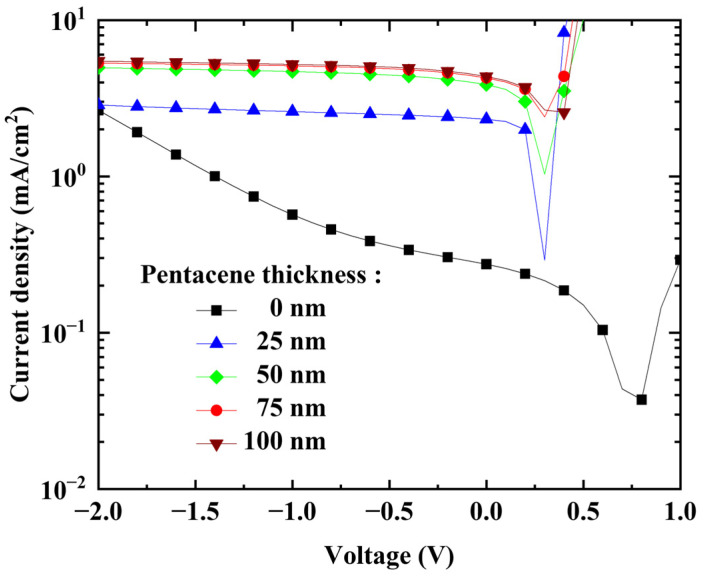
J–V characteristics with pentacene thickness measured under medium illumination level.

Under the illumination condition with the medium level (Figure 3), the photocurrent of the OPDI device was dependent on the thickness of the pentacene layer, as shown in Figure 7. When the pentacene was not used, the photocurrent density was 0.57 mA/cm^2^ under a reverse bias of −1 V, while its dark current was 0.215 mA/cm^2^. Upon increasing the thickness of the pentacene layer from (50 to 75) nm, the photocurrent increased from (4.68 to 5.1) mA/cm^2^, while the dark current densities were (0.014 and 0.011) mA/cm^2^, respectively. This is attributed to the increase in the amount of absorbed light on increasing the thickness of the absorbing pentacene layer. However, for the thickness of 100 nm, the photocurrent (5.2 mA/cm^2^) did not increase significantly (dark current density: 0.007 mA/cm^2^). This may be attributed to the trade-off between two opposing effects. One is the positive effect of the increase in light absorption on the increase in thickness. The other is the negative effect of the reduction in the extraction of photo-generated charge carriers and/or the increased probability of the recombination loss of excitons due to the longer diffusion path [42]. Thus, to optimize the OPDI performance, the thickness of the pentacene layer should be finely tuned.

**Figure 8:**
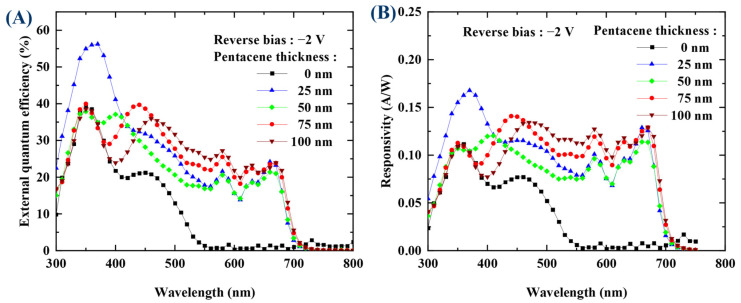
(**A**) External quantum efficiency and (**B**) responsivity with pentacene thickness measured under −2 V reverse bias.

Figure 8A shows the external quantum efficiency (EQE) spectra with different thicknesses of the pentacene layer. The EQE is defined as the ratio between the number of incident photons and the extracted number of the photo-generated free electrons and can be expressed as:

(1) $$EQE=\frac{I_{p}}{P_{in}}\frac{h}{q}$$

where *I_p_* is the photogenerated current, *P_in_* is the incident light intensity, *h* is Plank’s constant, *ν* is the frequency of light, and *q* is the electron charge.

The EQE in the wavelength range (500–700) nm increases when increasing the thickness of the pentacene layer. Exceptionally, the EQE with 25 nm pentacene exhibited a slightly higher EQE than that with 50 nm pentacene. We have conducted the experiment twice under the same condition with the pentacene thickness of 25 nm to verify the accuracy, and obtained exactly the same results, which are provided in Figure S1 of the Supporting Information (SI). According to the literature, the thickness dependence of the active layer on the photo-generated current can be attributed to a compromise between an increased absorption and a charge recombination loss due to the increase in the traveling distance of carriers with increasing the thickness of the active layer, the impact of grain size, and so on [43,44,45,46]. This paper addresses the surface morphology being affected by the grain size of the active layer, as a consequence affecting the dark current and photo-generated current. Accordingly, we have investigated the surface morphology with the thickness of the pentacene layer using secondary electron microscopy (SEM), the results of which are presented in Figure S2 of the Supporting Information (SI). To measure the surface morphology, the pentacene layer with the corresponding thickness was deposited on the glass/ITO (150 nm)/MoO_3_ (10 nm) layers. From the results, no grains were found on the ITO/MoO_3_ layers and the grain size was slightly increased in proportion to the evaporation time. Interestingly, the apparent grain boundaries appeared between grains somewhat differently depending on the thickness. All these factors will collectively influence the external quantum efficiency, depending on the thickness of the pentacene layer.

**Figure 9:**
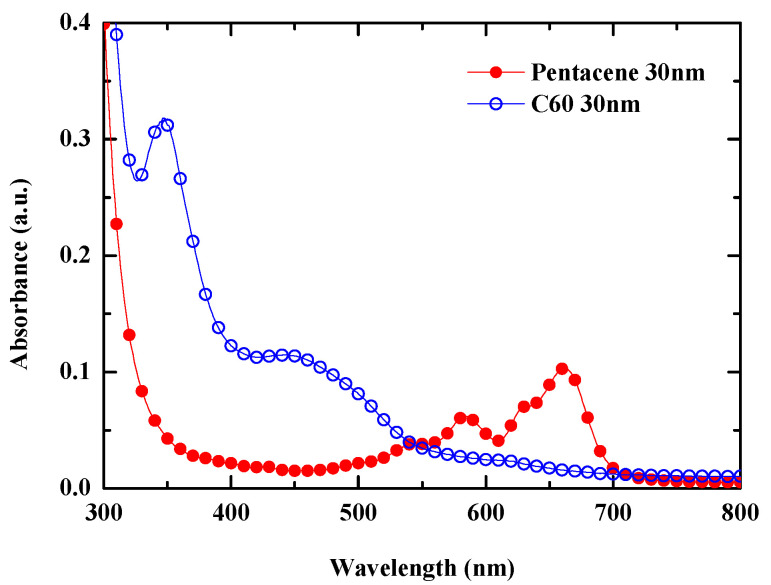
The measured absorption spectra of the pentacene and C_60_.

Note that the resonance wavelength is proportional to the thickness times of the effective refractive indices of the active layers between the electrodes [47,48]. Thus, by keeping the thickness of the C60 constant and increasing the thickness of the pentacene, the resonance wavelength also increases. This phenomenon can be seen clearly in Figure 9. The OPDI device without the pentacene layer shows nearly zero EQE in this range of (500–700) nm, which shows a non-resonance response. However, with the pentacene included, two EQE peaks appear in the wavelength range (350–500) nm. The first peak at (350–360) nm was almost fixed independent of the thickness of the pentacene, and the second peak, which is attributed to the resonance effect, moves to a higher wavelength upon increasing the thickness of the pentacene. When the thickness of the pentacene is 25 nm, the resonance wavelength nearly coincides with the wavelength of the maximum absorption peak without the cavity effect, resulting in absorption enhancement. That is, the EQE with the 25 nm pentacene was 56% at the wavelength of 360 nm, which was enhanced by 1.5 times compared to 39% at the wavelength of 360 nm, without the cavity effect. When the thickness of the pentacene was 75 nm, the second peak at the wavelength of 440 nm seemed to be enhanced to 40% by the second absorption peak (~21% EQE) present at the same wavelength of 440 nm without the cavity effect. The responsivity of the OPDI devices with a different thickness of the pentacene layer follows a similar trend to the EQE spectra, as shown in Figure 8B. The peak responsivity attributed to the 25 nm pentacene layer exhibited the value of 0.17 A/W at a wavelength of around 360 nm.

The responsivity is defined as the ratio of the photogenerated current to the power intensity of incident light and can be expressed as:

(2) $$R=EQE\frac{q}{hc}$$

where *λ* is the wavelength of light in nm and *c* is the speed of light in vacuum.

To analyze the EQE spectra, the absorption spectra of pentacene and C60 were investigated, and Figure 9 shows the results. It is noticeable that the pentacene shows light absorption in the wavelength range (550–700) nm with a maximum peak around 660 nm, which coincides with the EQE peak around that wavelength shown in Figure 8A. The absorption spectrum of C60 shows a strong absorption in the wavelength range (300–400) nm with a maximum peak around 350 nm, which coincides with the EQE peak at this position of Figure 8A. According to the literature [49], the HOMO–LUMO transition in C60 is forbidden; instead, the vibronic transitions are allowed via anharmonic vibrations, which results in the above gap transition in the range (200–540) nm. The absorption peak at a wavelength of around 350 nm in Figure 9 coincides with that reported in this paper. Thus, we can see that the EQE peak around 350 nm in Figure 8A is attributed to C60, while the EQE peak around 660 nm comes from the pentacene. Conclusively, the movement of the second peak from (400 to 450) nm with increasing the thickness of the pentacene layer is attributed to the optical cavity effect. That is, the resonant wavelength owing to the cavity effect increases as the cumulative optical path length increases with increasing the thickness of the pentacene layer. When the pentacene was not used, the EQE spectra resembled the absorption spectrum of the C60 of Figure 9. 

**Figure 10:**
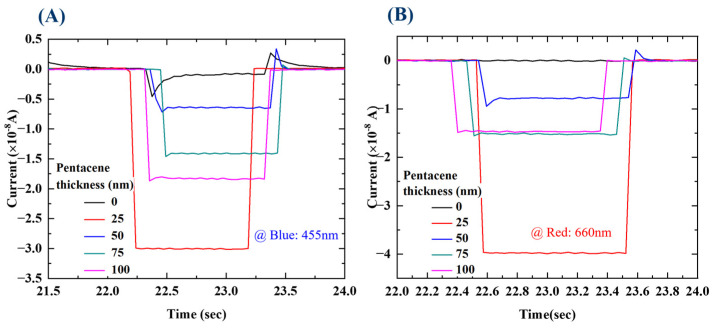
Normalized transient photocurrent measured under (**A**) 455 nm blue light and (**B**) 660 nm red light at 0.71 mW/cm^2^ and V = 0 V.

To further evaluate the OPDI performance, we performed more characterizations. Essentially, the fast response is important for various sensing applications of the OPDI. The rise and fall time is defined as the time interval for the photocurrent reaching from 10% up to 90% and 90% down to 10% of its maximum value. The OPDI with a pentacene layer thickness of 50 nm shows the shortest rise time of 34.1 ms and fall time of 34.25 ms under a blue (455 nm) light source at 0.71 mW/cm^2^, the results of which are shown in Figure 10, including the rise/fall times of 41.4/36.5 ms under red light (660 nm). This might be related to the diffusion time and the trap states depending on the active layer thickness and the morphology. 

Detectivity is another important parameter for the OPDIs’ applications, which can be evaluated using the calculation as D* = R/(2qJ_dark_)1/2 considering the shot noise from the dark current as the major contribution of total noise, where R is the responsivity, J_dark_ is the dark current, and q is the elementary charge. Thus, D* is the figure of merit to characterize the capability of weak light detection for the OPDI, considering both photoelectric conversion efficiency and noise current. The calculated detectivity is presented in Figure S3. It is seen that the detectivity increases from approximately 5 × 10^8^ to 1.5 × 10^9^ Jones, and the pentacene thickness increases from 25 nm to 75 nm. It decreases again at the higher thickness of 100 nm compared to that of 75 nm. The maximum detectivity was shown to be 1.45 × 10^9^ Jones at a 440 nm wavelength for the pentacene thickness of 75 nm.

In summary, we have presented work on the fabrication and characterizations of the fully small molecule-based organic photodiodes using the in-line vacuum evaporation method. With the conventional materials of pentacene as the donor and C60 as the acceptor, the EQE at a wavelength of 370 nm increased by 1.5 times via the resonance absorption enhancement, compared to the non-cavity device without the pentacene layer. The MoO_3_ and BCP played roles as an electron-blocking layer and hole-blocking layer, respectively, resulting in the effective suppression of the dark current. We expect further studies to focus on the performance improvements using the bulk heterojunction structure, and to extend the absorption wavelength to near-infrared (NIR) using other small molecules.

## 4. Conclusions

In summary, we present all small molecule-based organic photodiodes fabricated using the vacuum evaporation method, a process which can provide promising features such as a well-defined molecular structure, high purity, large scalability, and good process repeatability. As the donor and acceptor materials, the conventional pentacene and C60 were used. MoO_3_ and BCP played a good role as an electron-blocking layer and hole-blocking layer, respectively. In particular, the BCP layer worked as the HBL played a main role to suppress the dark current, of which the current density was reduced by two orders higher than that without the HBL.

By varying the thickness of the pentacene layer, the resonance wavelength was tuned via a cavity effect. By virtue of this effect, the light absorption at 360 nm, of which the wavelength corresponds to the maximum absorption of C60, was enhanced by 1.5 times, compared to that without the non-cavity device. To reduce the dark current and increase the responsivity, further study, such as the bulk heterojunction and the use of other small molecules, is needed, specifically to extend the absorption wavelength up to the near-infrared.

## Figures and Tables

**Table 1:** Comparisons of the dark current, on-current, and On/Off ratio, depending on the device type.

	Device Type	With Block Layers	w/o HBL	w/o EBL	w/o HBL and EBL
Current					
Dark current (−1 V reversed)		−0.01217 mA/cm^2^	−3.34673 mA/cm^2^	−0.06581 mA/cm^2^	−2.0675 mA/cm^2^
Medium illumination (−1 V reversed)		−6.73462 mA/cm^2^	−12.9272 mA/cm^2^	−4.42037 mA/cm^2^	−7.64342 mA/cm^2^
On/Off Ratio		×553.38	×3.86	×67.17	×3.70

## Data Availability

The data that support the findings of this study are available from the corresponding author upon reasonable request.

## Supplementary Materials

The following supporting information can be downloaded at: https://www.mdpi.com/article/10.3390/nano13212820/s1, Figure S1: EQEs for the OPDIs experimented twice with the pentacene 25 nm; Figure S2: the surface morphology measured by SEM with the thickness of pentacene layer on ITO/MoO_3_. Figure S3: The detectivity with Pentacene thickness measured under −2 V reverse bias.
