# Supplementary material for: Analyses of All Small Molecule-Based Pentacene/C60 Organic Photodiodes Using Vacuum Evaporation Method
Source: Nanomaterials (Basel). 2023 Oct 24;13(21):2820. doi: 10.3390/nano13212820 (PMC10650082; doi:10.3390/nano13212820)
Supplement: Supplementary file 1 [file nanomaterials-13-02820-s001.zip › nanomaterials-2646610-supplementary.pdf]

nanomaterials

MDPI

Supporting Information

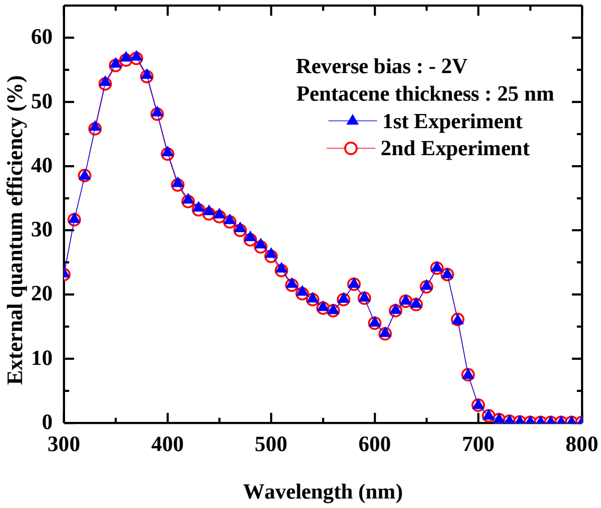

Figure S1. EQEs for the OPDIs experimented twice with the pentacene 25 nm

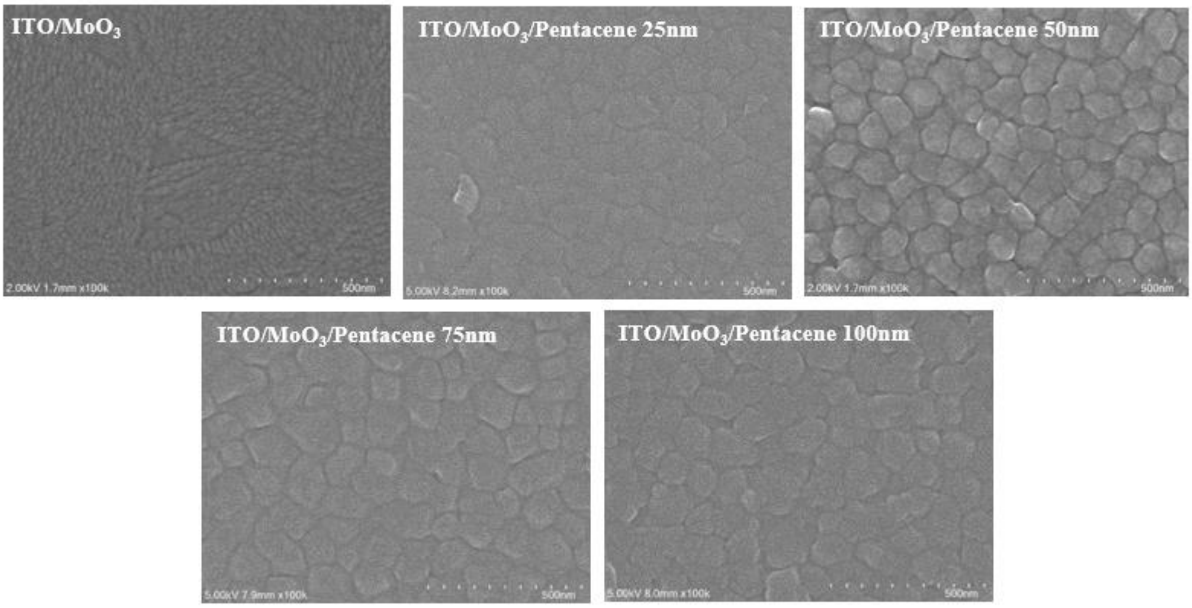

Figure S2. The surface morphology measured by SEM with the thickness of pentacene layer on ITO/MoO_3.

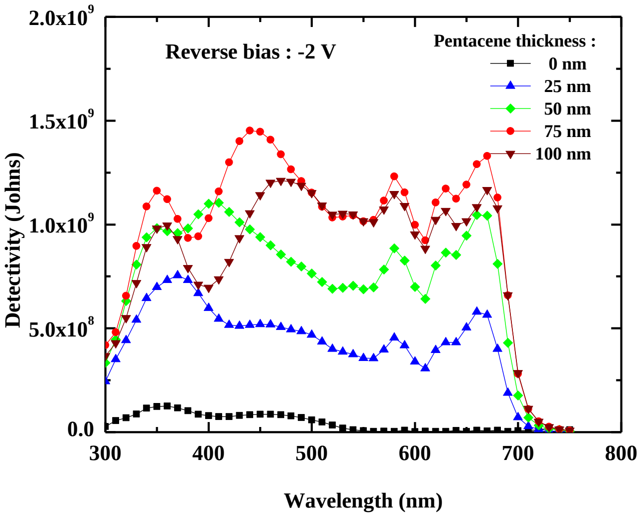

Figure S3. The detectivity with Pentacene thickness measured under −2 V reverse bias.
